# The Brain Observatory Storage Service and Database (BossDB): A Cloud-Native Approach for Petascale Neuroscience Discovery

**DOI:** 10.3389/fninf.2022.828787

**Published:** 2022-02-15

**Authors:** Robert Hider, Dean Kleissas, Timothy Gion, Daniel Xenes, Jordan Matelsky, Derek Pryor, Luis Rodriguez, Erik C. Johnson, William Gray-Roncal, Brock Wester

**Affiliations:** Research and Exploratory Development Department, Johns Hopkins University Applied Physics Laboratory, Laurel, MD, United States

**Keywords:** connectome, software, cloud, data, storage, imaging, electron microscopy, X-ray

## Abstract

Technological advances in imaging and data acquisition are leading to the development of petabyte-scale neuroscience image datasets. These large-scale volumetric datasets pose unique challenges since analyses often span the entire volume, requiring a unified platform to access it. In this paper, we describe the Brain Observatory Storage Service and Database (BossDB), a cloud-based solution for storing and accessing petascale image datasets. BossDB provides support for data ingest, storage, visualization, and sharing through a RESTful Application Programming Interface (API). A key feature is the scalable indexing of spatial data and automatic and manual annotations to facilitate data discovery. Our project is open source and can be easily and cost effectively used for a variety of modalities and applications, and has effectively worked with datasets over a petabyte in size.

## 1. Introduction

Mapping the brain to better understand cognitive processes and the biological basis for disease is a fundamental challenge of the BRAIN Initiative. Technological advances in neuroimaging have grown rapidly over the last ten years, making it almost routine to image high-resolution (sub-micron) brain volumes in many laboratories around the world using Electron Microscopy (EM) and X-Ray Microtomography (XRM), among other imaging modalities (Bock et al., 2011; Helmstaedter et al., 2013; Kasthuri et al., 2015; Lee et al., 2016; Dupre and Yuste, 2017; Witvliet et al., 2021). These datasets, which provide the means to resolve individual neurons and the individual connections (synapses) between them, are highly valuable for providing key insights into neural connectivity and neuroanatomical features. As these high resolution neuroimaging volumes grow in extent, however, substantial challenges have emerged, including efficient data storage, the computational and financial cost of indexing and querying, and the technical difficulty of big-data visualization (Helmstaedter et al., 2013; Lichtman et al., 2014).

As new tools for interrogating neuroimaging datasets at high resolutions advance and become more common, a centralized data-access and data-processing paradigm is needed in order to take advantage of economies of scale when operating at the tera- to petascale level. While research groups are beginning to embrace data archives, most treat the system as simply a place to deposit finalized data, with raw datasets generated and stored in a custom format and analyzed and inspected with custom software. At increasing data scale, it is quickly becoming impossible for researchers to characterize many of the underlying properties. For many recently-generated image volumes approaching the petascale, it is likely that most of the dataset is never viewed in detail by a human. Additionally, conventional approaches for automatically or semi-automatically reconstructing neuronal maps focus on building methods for small volumes, and scaling these tools to operate on multi-terabyte or petabyte data volumes, is often significantly beyond the capabilities and budgets of a single research group.

Large datasets are incredibly rich in scientific content which should be shared with others to best leverage the investment of time and resources, and to fully exploit the value of the data. Due to the challenges in collection, storage, and analysis of terascale and petascale data volumes, few public datasets of this size are routinely shared, even though many such volumes exist on local, private storage, and many petabytes of new data are anticipated in the future from programs like the BRAIN Initiative and other future large scale programming (Mikula, 2016; Dorkenwald et al., 2019; Wilson et al., 2019; Morgan and Lichtman, 2020; Scheffer et al., 2020; Phelps et al., 2021; Witvliet et al., 2021).

**Figure 1:**
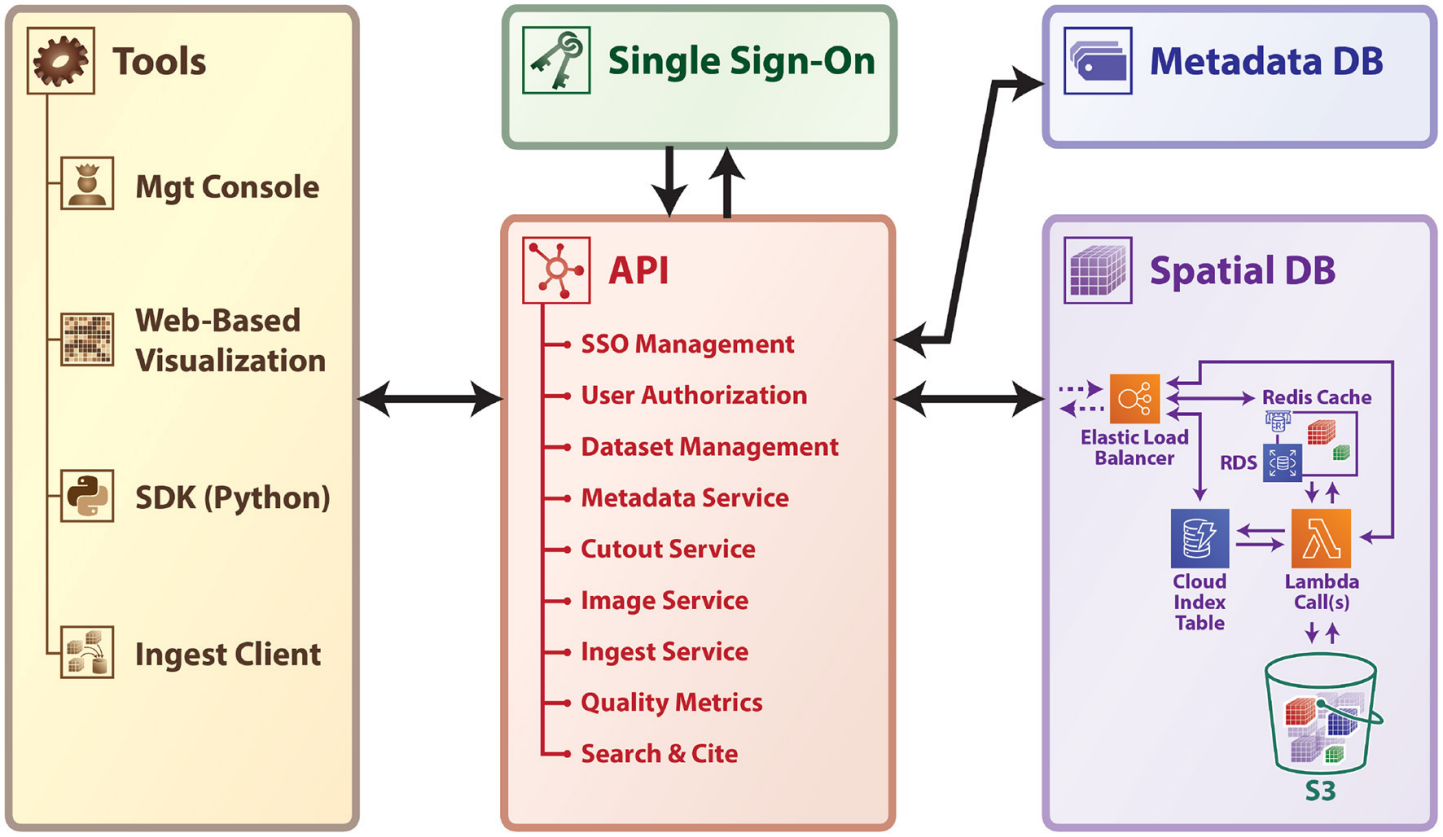
A high-level schematic of BossDB platform.

We considered use cases such as the first fully-automated pipelines for processing and assessing XRM (Dyer et al., 2017) and EM datasets (Bock et al., 2011; Kasthuri et al., 2015; Lee et al., 2016) and work by many academic laboratories around the world to understand state-of-the-art approaches and their limitations. We emphasize that high-performance and scalable data storage is an essential component of any modern connectomics effort, due to the need for rapid, multi-user data access. In designing our Brain Observatory Storage Service and Database (BossDB), we researched several related efforts, including DVID^1^ (Katz and Plaza, 2019) which excels in versioned terascale storage; CATMAID and Knossos (Saalfeld et al., 2009; Helmstaedter et al., 2011) which provide a mature manual annotation platform. We previously worked with NeuroData to develop ndstore (Burns et al., 2013), which originated and implemented many of the design principles necessary to store and access high-dimensional imaging datasets. These principles include (1) an efficient internal data representation and associated spatial indexing scheme; (2) an API to remotely access services; and (3) MATLAB and Python toolkits to facilitate usability. Based on this prior research and an understanding of the evolving requirements driven by new and maturing imaging modalities, we created a robust, cloud-native petascale datastore with a number of services and support tools (Figure 1).

## 2. Methods

To enable large-scale, collaborative research we developed and deployed a cloud-native data archive to support the storage, analysis, and sharing of large spatial datasets. Service-oriented architectures have continued to grow in popularity and possess many appealing properties when designing a cloud-based data archive (Vogelstein et al., 2016). Our solution, BossDB, is deployed within the Amazon Web Services (AWS) ecosystem and has been robustly designed to leverage cloud capabilities and ensure a highly-available, scalable, and cost-efficient system. Other research teams have previously deployed their own instantiations of BossDB (Vogelstein et al., 2016; Dyer et al., 2017).

### 2.1. Spatial Database

The spatial database is the foundation of BossDB, and uses the strengths of the cloud to efficiently store and index massive multi-dimensional image and annotation datasets (i.e., multi-channel 3D image volumes). A core concept is our managed storage hierarchy, which automatically migrates data between affordable, durable object storage (i.e., Amazon Simple Storage Service or S3) and an in-memory data store (i.e., Redis), which operates as read and write cache database for faster IO performance with a tradeoff of higher cost. The BossDB cache manages a lookup index to determine the fastest way to return data to the user, taking advantage of data stored in the hierarchy. While this requires the use of provisioned (non-serverless) resources, this allows for storage of large volumes at a low cost, while providing low latency to commonly accessed regions. We utilize AWS Lambda to perform parallel IO operations between the object store layer and memory cache layer and DynamoDB for indexing. These serverless technologies allow BossDB to rapidly and automatically scale resources during periods of heavy operation without incurring additional costs while idle.

**Figure 2:**
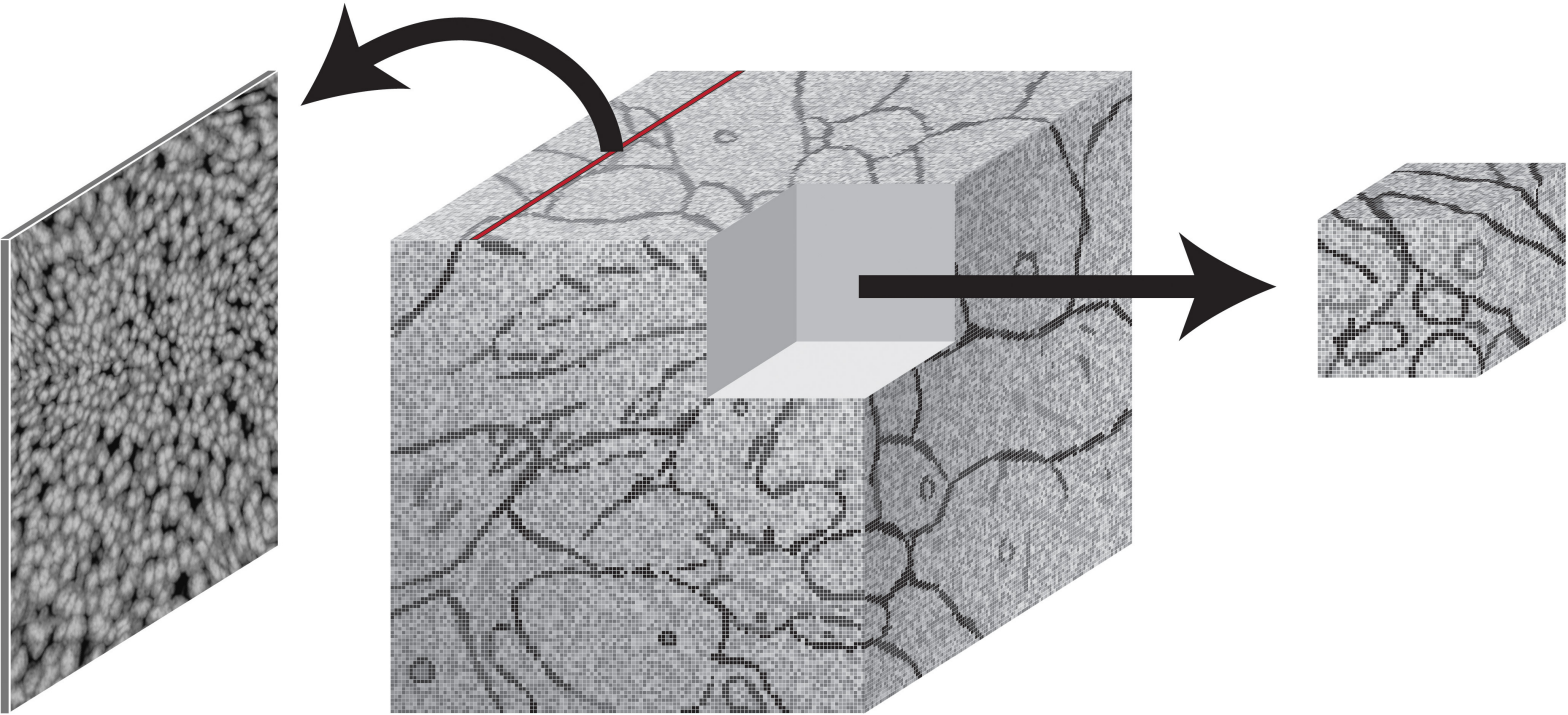
An illustration showing image slices (left) being composed into 3D cuboid volumes (middle). Arbitrary requests may be made to extract image regions of interest (right).

**Figure 3:**
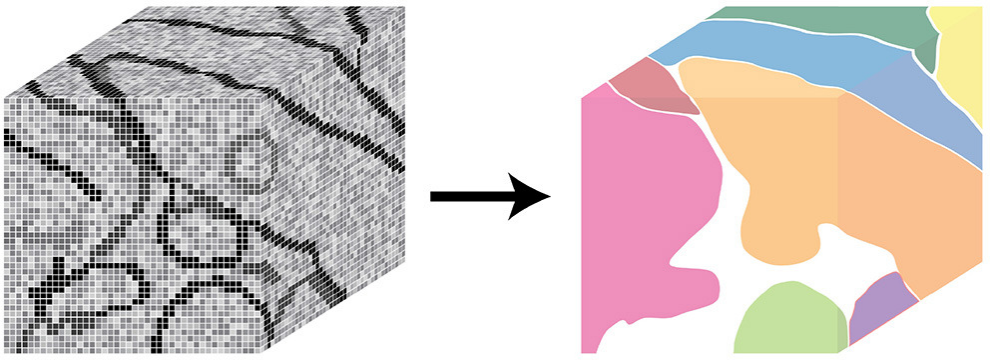
An illustration showing *annotations*, composed of voxel labels (left) and how a unique annotation identifier can represent a unique *object* in the image data (right).

**Figure 4:**
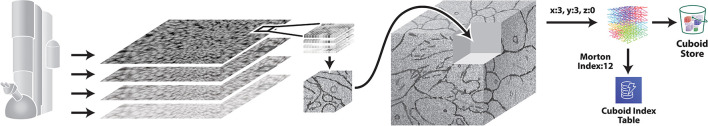
An illustration showing how large 2D image slices generated by EM imaging are re-formatted as cuboids, which fit into a larger 3D volume, indexed using a *z*-order curve.

The BossDB spatial database is designed to store petascale, multi-dimensional image data (i.e., multi-channel three-dimensional image volumes, with optional time series support, Figure 2) and associated coregistered voxel annotations (Figure 3). In this context, voxel annotations are unsigned 64-bit integer (uint64) labels stored in a separate *channel* that is in the same coordinate frame as the source image data. Each unique uint64 value represents a unique *object* (e.g., neuron, synapse, organelle). A user can leverage annotations within various channels (e.g., “segmentation,” “mitochondria”) to create groups of voxels to define objects that have some semantic meaning, typically the result of manual annotation or automated processing. The database maintains an index of annotation locations, enabling efficient spatial querying and data retrieval (Figure 4).

The internal representation of volumetric data utilizes small cuboids, or 3D chunks of data (i.e., 512 × 512 × 16 voxels, which can vary in dimension), which are stored in Amazon S3 as compressed C-order arrays. Cuboids are indexed using a Morton-order space-filling curve, which maps the 3D location of each cuboid to a single dimension. In addition, annotations are indexed so BossDB can quickly retrieve which annotation IDs exist in an individual cuboid, and in which cuboids a unique ID exists. With these indices, all of which are stored in auto-scaling Amazon DynamoDB tables, the BossDB API can provide spatial querying of annotations by ID and efficient retrieval of arbitrary data volumes. The database will also render and store a resolution hierarchy through downsampling of a dataset, which is critical for visualization applications to efficiently provide low-resolution views and useful when processing large datasets. The spatial database supports various bit-depths (including uint8, uint16 image channels and uint64 annotation channels) and we will provide additional bit-depth and data formats as needed.

Additionally, BossDB is able to store various mesh files associated with voxel annotation channel ID values, including precomputed format (Maitin-Shepard, 2021), which can be accessed through our API by visualization applications.

### 2.2. Single Sign-On Identity Provider

A centralized and standalone authentication server provides single sign-on functionality for BossDB and integrated tools and applications. This allows BossDB to control permissions internally and operate securely, while maintaining the ability to federate with other data archives in the future.

We use the open source software package Keycloak as an identity provider to manage users and roles. We created a Django OpenID Connect plugin to simplify the integration of services with the SSO provider.

Our identity provider server intentionally runs independently from the rest of BossDB system, forcing the BossDB API to authenticate just like any other SSO integrated tool or application, and making future federation with other data archives or authentication systems easy. The Keycloak server is deployed in an auto-scaling group that sits behind an Elastic Load Balancer in order to achieve high-throughput database requests with minimal latency.

### 2.3. Application Programming Interface

As the primary interface to BossDB, the API provides a collection of versioned, RESTful web services. It enforces access permissions and organizes data in a logical data model for spatial and functional results. Because the API is versioned, the BossDB storage engine can support significant changes while still maintaining backwards compatibility with legacy applications and tools. This BossDB API was designed from first principles to be versioned, and so this feature adds little in the way of day-to-day engineering complexity. All requests to the API are authenticated through the SSO service or via a long-lived API token, which enables tracking usage and throttling requests as needed to manage cost and ensure reliable performance (e.g., high bandwidth power user vs. a limited guest user). The services BossDB provides are summarized below:

#### 2.3.1. SSO Management and User Authorization

A set of services to manage users, roles, groups, and permissions. Roles limit what actions a user can perform on the system, while permissions limit what data users can access or manipulate. Permissions are applied to BossDB datasets via groups, making it easy to manage and control access for both individuals and teams. Through the application of permissions, a researcher or administrator can choose to keep a dataset private, share with collaborators, or make it publicly available.

#### 2.3.2. Dataset Management

The BossDB API organizes data into a logical hierarchy to group related data together (e.g., source image data and associated annotations, 2-photon and EM datasets from the same tissue sample). This service provides interfaces to create and manage datasets and their properties.

#### 2.3.3. Ingest Service

A critical challenge when using a centralized data archive is the ingest of large datasets to standardized formats from diverse local storage formats and organization paradigms. The Ingest Service enables the moving of large datasets of varying data formats (Table 1) from local or cloud storage into BossDB by performing the upload of data into the cloud and then ingesting that data into the spatial database format, allowing independent scaling and failure recovery. The service provides methods to create a new ingest job, monitor the status of a job, join an upload client worker to a job, and cancel a job. Unlike general upload tools that run on client-side compute infrastructure, or commands like the *aws* command-line offerings that may run on a single host, the ingest client is able to perform ingests on arbitrarily many compute nodes, with graceful error management even in the case that a compute node powers down during an ingest job.

**Table 1:** Data types and associated data formats that are supported by tile and chunk/volumetric based ingest service processes.

**Data type**	**Data format**	**Ingest type**
JPEG	8-bit, 16-bit	Tile
PNG	8-bit, 16-bit	Tile
TIFF	8-bit, 16-bit	Tile
CATMAID	Native format	Tile
HDF5	Any encoding	Tile/Chunk
N5	Any encoding	Tile/Chunk
Zarr	Any encoding	Tile/Chunk
CloudVolume	Native format	Tile/Chunk
DVID	Native format	Tile/Chunk
Nifti	Any encoding	Tile/Chunk
Dicom	Any encoding	Tile/Chunk
Knossos	Any encoding	Tile/Chunk

##### 2.3.3.1. Tile Ingest

**Figure 5:**
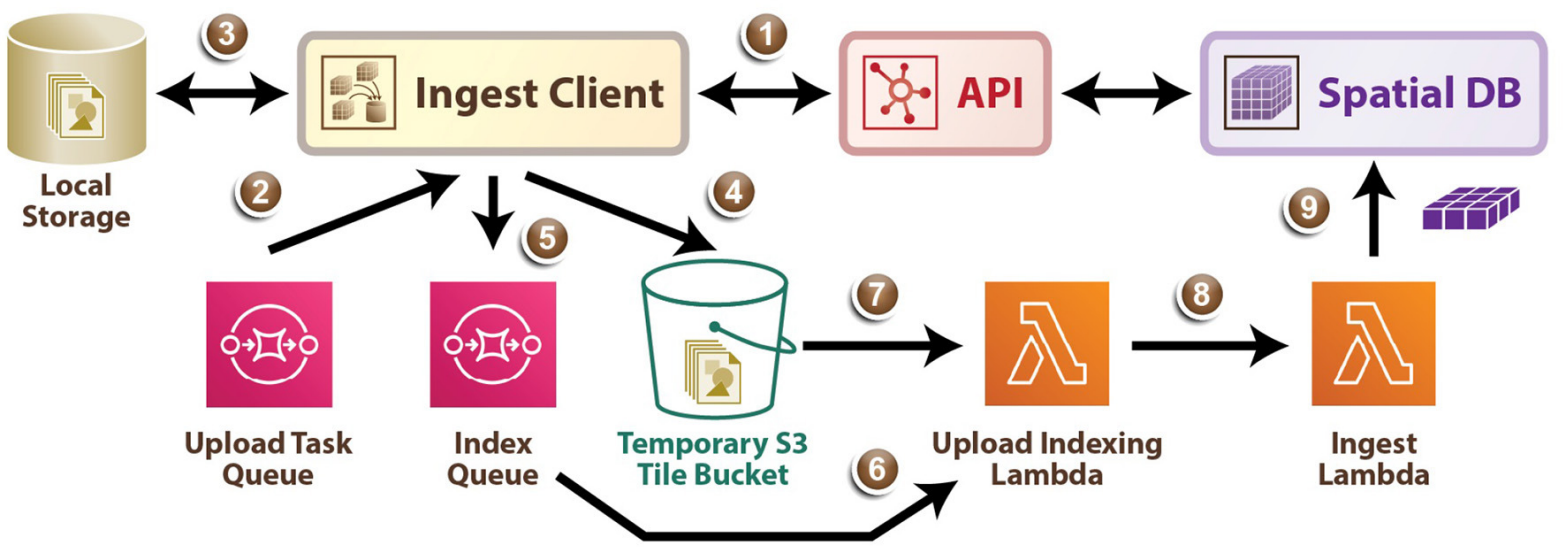
A diagram outlining the data ingest service process.

As demonstrated in Figure 5, the ingest process directly leverages AWS infrastructure, scaling on demand. First, using the ingest client a user uploads an ingest job configuration file to the API (1) which populates a task queue, enumerating all tiles that must be uploaded, and returns temporary AWS credentials. Next, the ingest client retrieves a task from the Upload Task Queue (2), and loads the requested local file into memory as an image tile (3), and uploads the tile data to an S3 bucket (4). The ingest client then writes a message to the index queue signaling it is finished with this tile (5). An AWS Lambda automatically fires when a message enters the Index Queue and it uses DynamoDB to track which tiles are successfully written to the tile bucket (6), (7) and when enough tiles in a region have arrived to generate the BossDB cuboid data representation, a second Lambda function is triggered (8). This Ingest Lambda function then loads the specified tiles, reformats them into cuboids, inserts them into the Spatial DB S3 bucket, updates the Spatial DB cuboid index, and finally marks the temporary tiles for deletion (9). The ingest client supports both parallel and distributed operation, allowing users to maximize their network bandwidth, especially in the case where source data is organized into numerous small image files.

##### 2.3.3.2. Volumetric Ingest

The ingest process also supports uploading three-dimensional chunks of data in the CloudVolume format^2^; this interface can be straightforwardly extended to other formats. Similar to Tile Ingest, the ingest client is used to upload an ingest-job configuration file to the API, populating a task queue with all chunks to be uploaded. The ingest client then retrieves a task from the Upload Task Queue, and loads that chunk into memory. The memory chunk is divided into multiple BossDB cuboids (512 x 512 x 16) and each cuboid is uploaded to an AWS S3 bucket. Upon uploading, the S3 update will trigger an AWS Lambda that copies the cuboid into main s3 store, adds an entry in DynamoDB, and marks the original cuboid for deletion.

#### 2.3.4. Dataset Metadata

BossDB can store arbitrary key-value pairs linked to data model items, which is useful to track experimental metadata and provenance (e.g., voxel size, animal information, annotation algorithm used). This service provides an interface to query, create, update, and delete key-value pairs associated with a dataset.

#### 2.3.5. Cutout Service

BossDB provides the *cutout service*, which enables users to interact with the Spatial Database by reading and writing arbitrary data volumes. While BossDB stores all data internally using a standardized format, the cutout service uses HTTP content negotiation to determine the data format of a request, allowing users to request specific database-supported formats when downloading data (e.g., compressed C-order blob, hdf5 file, pickled numpy array). The same is true of data-uploads: A user-provided content annotation enables BossDB to accept data in a variety of volumetric and image-based formats. This service enables scalable analytics by letting users access arbitrary chunks of data in parallel, perform automated processing, and write the annotation result back to BossDB. It also supports querying for the spatial properties of annotations, such as the bounding box of an annotation or identifying which annotations exist within a region.

#### 2.3.6. Image Service

In addition to our volumetric cutout service, we provide an image service to meet common user needs, which retrieves a 2D slice of data from the spatial database along one of the three orthogonal planes (i.e., XY, XZ, YZ), encoded as an image file. Again, HTTP content negotiation is used to determine the format of the response (e.g., png, jpeg). The service supports arbitrary image sizes or a fixed tile size, which is often used by visualization tools.

#### 2.3.7. Downsample Service

To allow users to quickly assess, process, and interact with their data, BossDB iteratively builds a resolution hierarchy for each dataset by downsampling the source data. This is a workflow that is run infrequently and on-demand, and needs to scale from gigabytes to petabytes of data. We developed a serverless architecture built on AWS Step Functions to manage failures and track process state. AWS Lambda is used to perform the underlying image processing in a parallel, scalable fashion. This approach helps to minimize costs since resources are only provisioned when needed and scale on-demand in a fully-automated paradigm. It is also possible to perform a partial downsample when only a portion of the original dataset has changed, saving the time and expense of re-running the process on the entire dataset. Image volumes with anisotropic native voxel sizes (e.g., x = 4 nm, y = 4 nm, z = 40 nm) are downsampled in the image plane dimensions (e.g., downsampling factors of x = 2, y = 2, z = 1) until block sizes reach near-isotropy (e.g. third downsample to resolution of x = 32 nm, y = 32 nm, z = 40 nm), after which they are downsampled equally in all dimensions. This remaining anisotropy diminishes higher in the downsampled hierarchy. In general, these levels are used primarily for visualization, and most analyses are performed at native or near-native resolutions (resolution 0 or 1).

### 2.4. User Tools

User facing tools are required to make a data archive truly useful, easy to use, and well documented. We currently offer a web-based management console, an ingest client, and a client-side Python module called intern for programmatic interaction^3^ (Matelsky et al., 2021). We have also integrated 3rd-party web-based data visualization tools. While BossDB API provides a rich interface to interact with the system, user friendly tools built on top of the API are important to increase utility and adoption by the community. We expect to mature and expand the scope of this tool library as community users build on the core BossDB technologies.

#### 2.4.1. Web-Based Management Console

BossDB has a web interface that lets users perform common actions interactively in their browser (e.g., create a dataset, monitor an ingest job, share a dataset with a user). This Django-backed web application is the primary interface for most users and will expose much of the API's functionality through an intuitive graphical interface. From the console, a researcher is able to manage datasets, discover new data, and launch the visualization tool.

#### 2.4.2. Web-Based Visualization

A critical capability to any data archive is the ability to easily visualize stored data. Whether inspecting ingested data, exploring a dataset, or sharing an interesting sample with a collaborator, the most common interaction with stored data will be through visualization. We integrated a version of Neuroglancer (Maitin-Shepard, 2021) to let users visually explore data stored in BossDB, and enable other visualization methods that provide abstraction over much of the API's complexity. The Neuroglancer interface may be used on all modern browsers and operating systems that support WebGL, including (as of the time of publication) Chrome version 51 or greater, Firefox version 46 or greater, and Safari 15.0 or greater. Through use of the imagery API, BossDB also supports mobile-friendly data visualization tools such as *Substrate* (Matelsky et al., 2020).

#### 2.4.3. Immersive Visualization and Annotation

The BossDB volumetric API likewise supports 3D collaborative annotation through immersive virtual reality (VR) tools such as syGlass (Pidhorskyi et al., 2018), which can enable high-throughput annotation of large volumes of dense imagery. VR takes advantage of the natural parallax of stereoscopic vision, which can improve the visual perception of complex 3D structures.

#### 2.4.4. Ingest Client

We have developed an open source ingest client in Python to manage uploading data to BossDB. The ingest process operates on a upload task queue which contains tasks specifying individual 2D tiles or 3D chunks of data to upload. To deal with the unique formats and file organization methods of diverse users, the client uses a simple plug-in design to import custom snippets of code responsible for taking a task, finding the right file, and loading the data into memory, which is then uploaded by the client. The work queue design allows copies of the client to be run distributed across compute nodes and in parallel on a single machine, substantially increasing throughput.

#### 2.4.5. Python Software Development Kit (SDK)

To support developers and researchers who want to programmatically interact with BossDB, we developed a pip-installable Python library that provides abstraction over much of the complexity in the API. Data cutouts of arbitrary size can be efficiently retrieved from our archive, enabling easy integration with analytics tools. The current SDK, called intern, will continue to be expanded and supported to accommodate updates and additions to the existing BossDB system and user requests.

## 3. Results

### 3.1. Motivating Application

Many of our design requirements for the BossDB ecosystem were motivated by the activities planned for the Intelligent Advanced Research Projects Activity (IARPA) Machine Intelligent from Cortical Networks (MICrONS) Program^4^. This effort seeks to enable the rapid advancement of artificial intelligence capabilities by creating novel machine learning algorithms that use neurally-inspired architectures and mathematical abstractions of the representations, transformations, and learning rules employed by the brain^4^. To guide the construction of these algorithms, the program centers around massive co-registered functional (e.g., two-photon calcium imaging) and structural (e.g., EM) neuroimaging experiments aimed at estimating the synapse-resolution connectome of a 1*mm*^3^ volume of mouse visual cortex, represented by nearly a petabyte of image and segmentation data, and using that information to constrain machine learning architectures. Our goal was to organize, store, and support the analysis of these large functional and anatomical datasets, and eventually enable public dissemination.

### 3.2. Deployment

**Figure 6:**
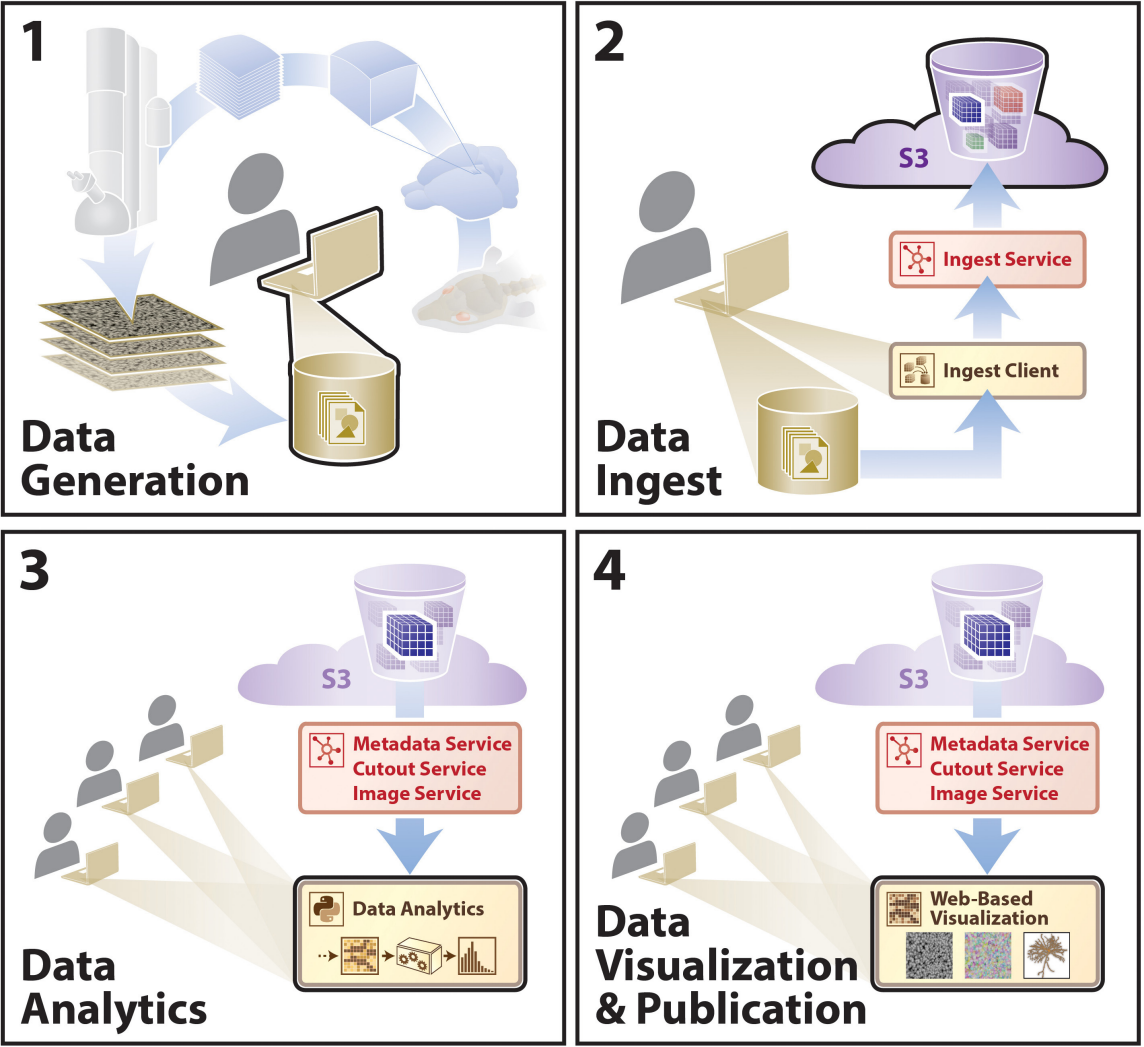
A diagram outlining an example user story showing utilization of the BossDB infrastructure. A typical research group collecting data for a hypothesis will move sequentially from (1)–(4). Other groups will extend these analyses using steps (3) and (4). Sample data included for demonstration (see text footnote 4).

We envision that this data archive will facilitate neuroscience inquiries through extensible, scalable processes, with a sample workflow outlined that includes data generation, data ingest, intra- and cross-dataset analysis, and multi-user data visualization in various workflows (e.g., data proofreading) outlined in Figure 6. During the IARPA MICrONS Program, a deployed instance of our BossDB system enabled concurrent proofreading operation by dozens of users, as well as the storage of a highly-available contiguous image volume that approached 2 PB of lossless EM image data (Bishop et al., 2021) using the *blosc* compression standard^5^. In addition to EM and segmentation datasets from the IARPA MICrONS program (https://bossdb.org/project/microns-minnie, https://bossdb.org/project/microns-pinky), we currently publicly store highly-available data for over 30 large-scale volumetric image collections, with multiple contiguous image volumes exceeding 100 TB in size (https://bossdb.org/projects/).

#### 3.2.1. Implementation

**Figure 7:**
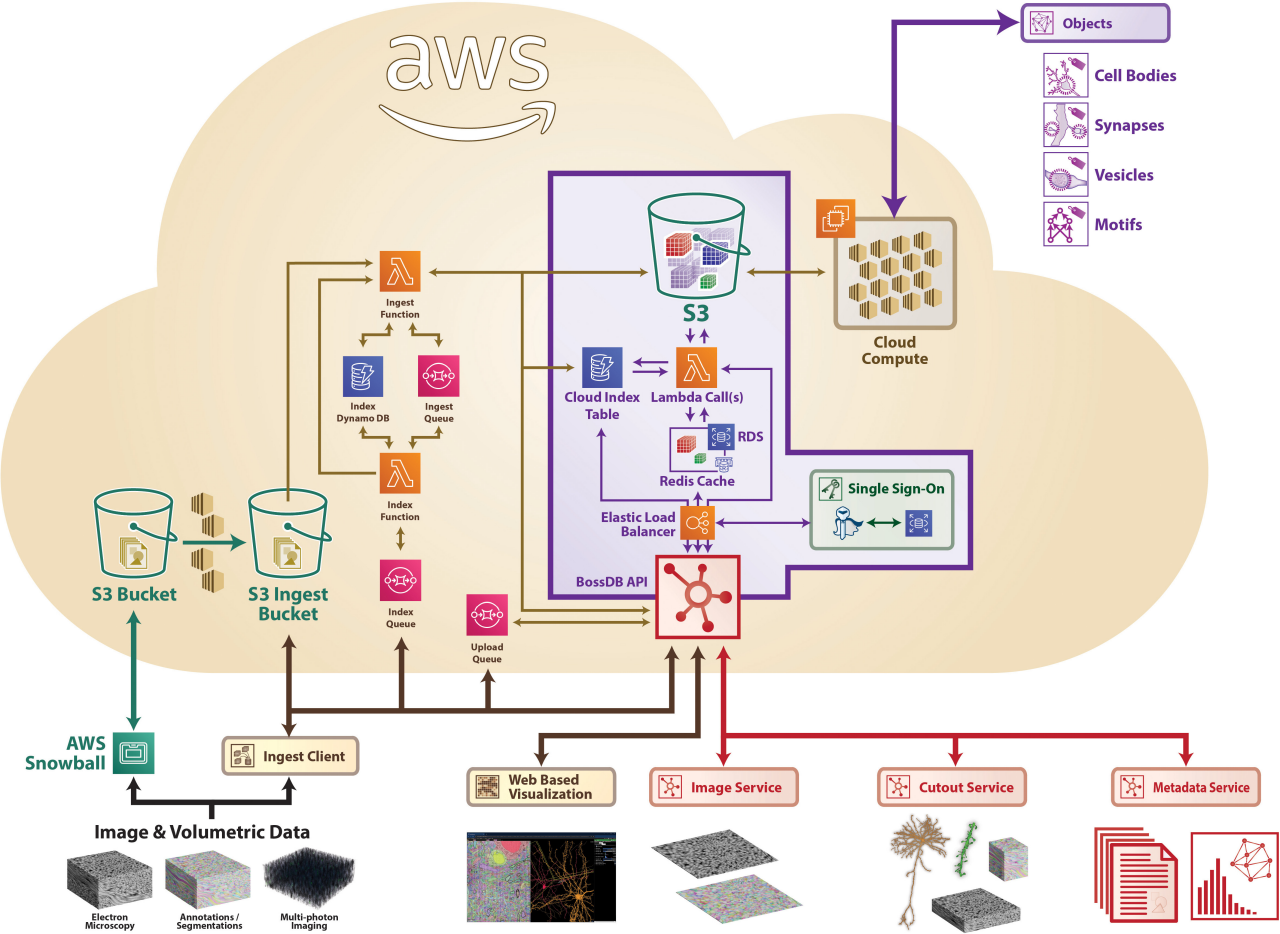
A high-level architecture diagram of BossDB as deployed using Amazon Web Services architecture, highlighting a number of services, including ingest processes on the left. Sample data included for demonstration (see text footnote 4).

Figure 7 shows the architecture of BossDB. The system has two user facing services: Authentication and Web Server Endpoint, both of which sit behind AWS elastic load balancers. The system uses Keycloak servers in a high-availability configuration for single sign-on authentication. The web server endpoints use Django API, to provide access to the majority of the services in BossDB.

BossDB uses serverless computing and storage, with AWS Lambda, SQS, S3, and DynamoDB to provide all of the other services mentioned in Section 2: Ingest, Metadata, Cutout, Image, and Downsample. Using serverless computing and storage for these components will automatically scale with demand and eliminate the need to maintain components.

BossDB is installed using the AWS CloudFormation service along with Salt and Packer to manage our infrastructure. This allows us to quickly duplicate the environment for testing and development and even change instance sizes within the new environments.

#### 3.2.2. Data Generation

**Figure 8:**
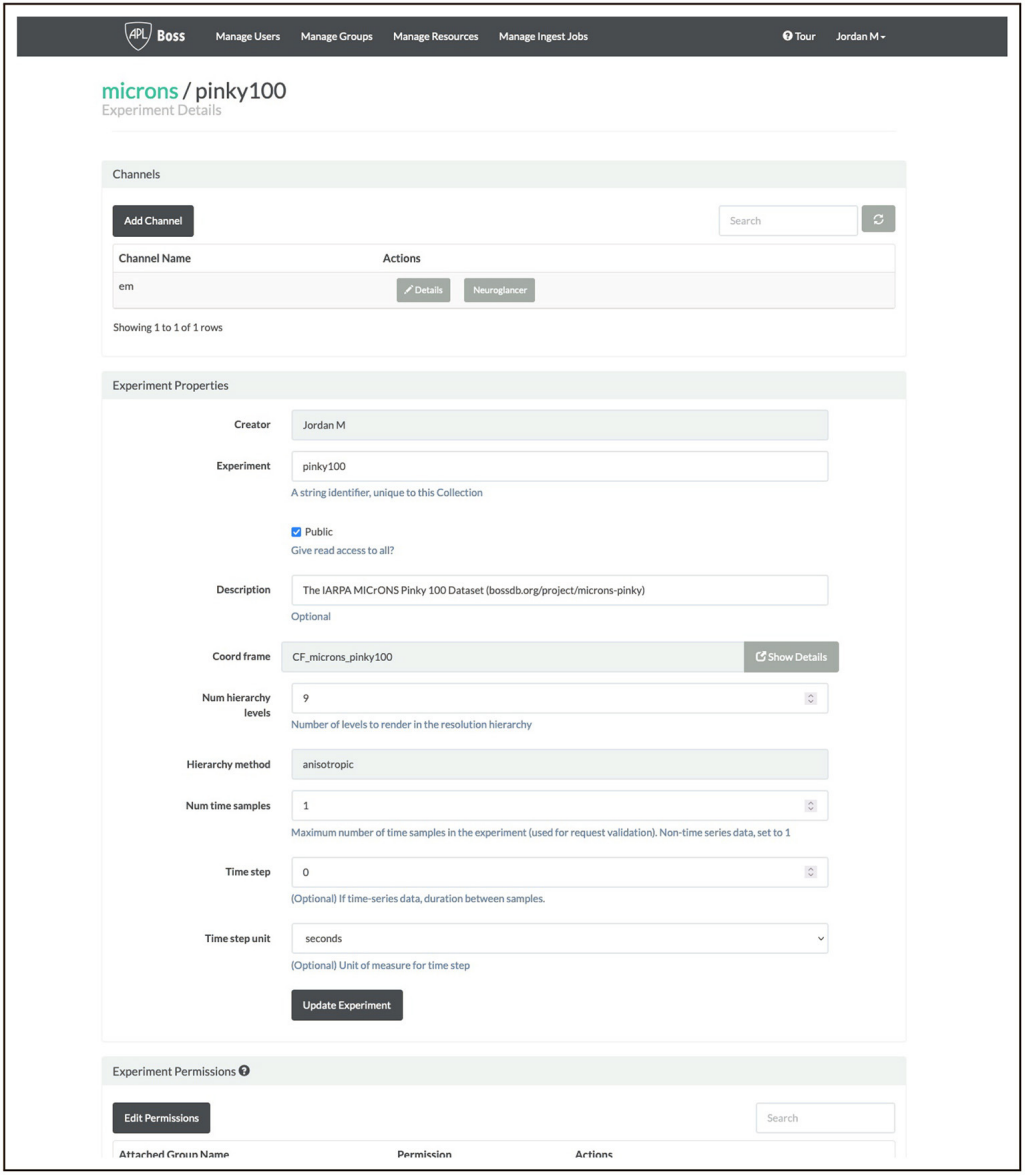
An example screenshot from our BossDB console for the MICrONS Pinky dataset (see text footnote 4).

Researchers collect experimental data; stitching, alignment, and registration take part prior to upload to BossDB. Users create new resources in BossDB to identify and store their datasets, recording their experimental parameters and dataset properties (e.g., voxel dimensions, bit depth, spatial extent) prior to upload. An example screenshot from our web console is shown in Figure 8; this setup can be accomplished programmatically using intern as well.

#### 3.2.3. Data Ingest

Once available, a researcher uploads image data via one of several methods supported by BossDB (e.g., REST API, ingest client), safely and efficiently storing data in BossDB. Large datasets can be uploaded incrementally, with data available for read as soon as it has been ingested, providing access to collaborators in minutes, not months.

The ingest client has already been used to upload petabytes of EM and calcium imaging data; many of these uploads proceed without any intervention from the developer team with the system automatically scaling to meet user's needs.

**Figure 9:**
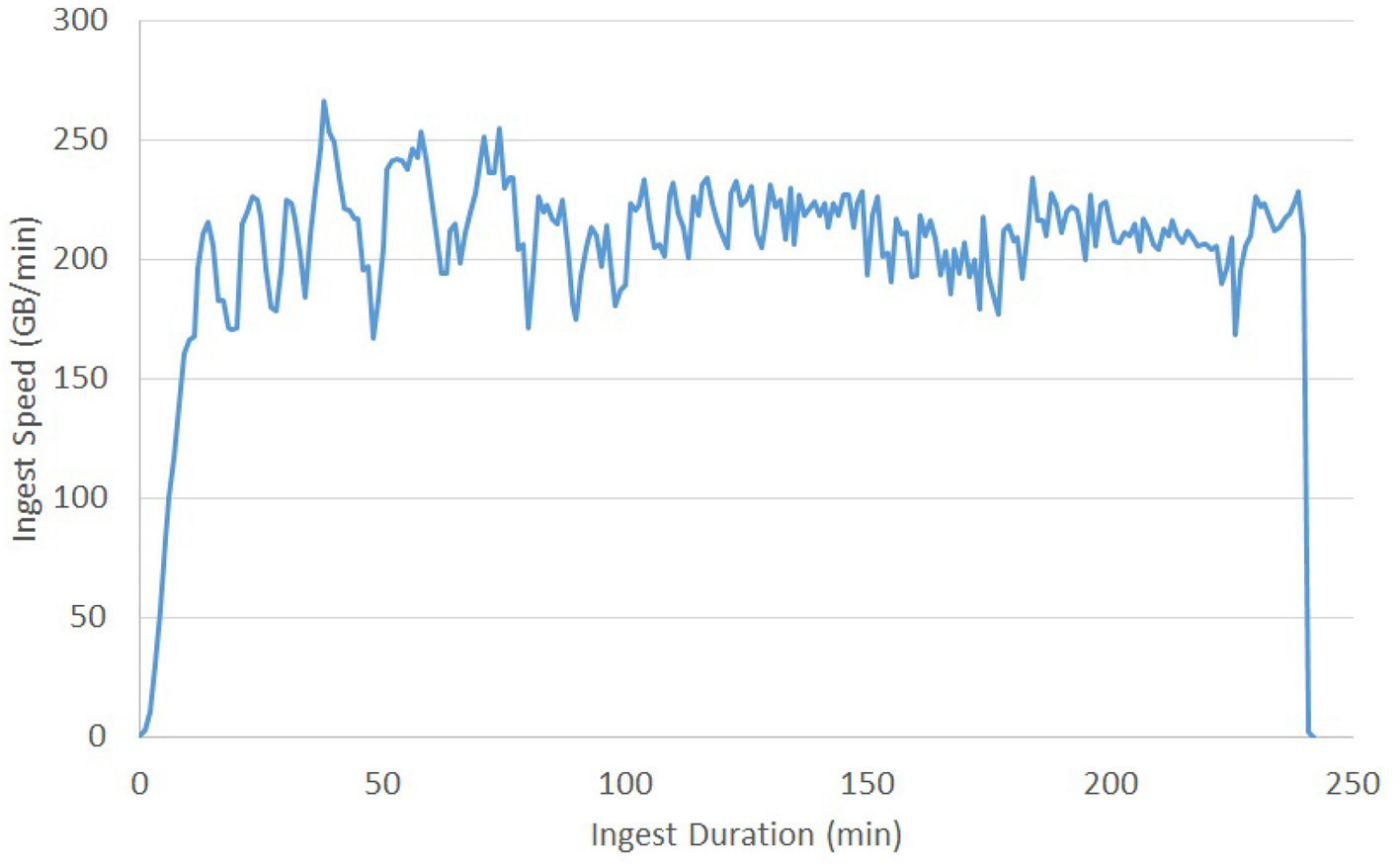
Volumetric Ingest throughput demonstrated over the complete ingest of a 50TB dataset in about 4 h.

Previous testing of the ingest process reached a sustained ingest throughput of 230 GB/Min (Figure 9) using the volumetric ingest-client into BossDB. The ingest client was run on 750 kubernetes pods across eight large servers uploading data from an AWS Bucket. AWS Lambda scaled to over 5000 concurrent executing functions to handle the load.

To perform at this speed we were running 12 Endpoint servers sized with m4.2xlarge instances, an RDS database backed with a db.m4.xlarge instance, and DynamoDB table sized at 2,000 read / 4,000 write capacity.

**Figure 10:**
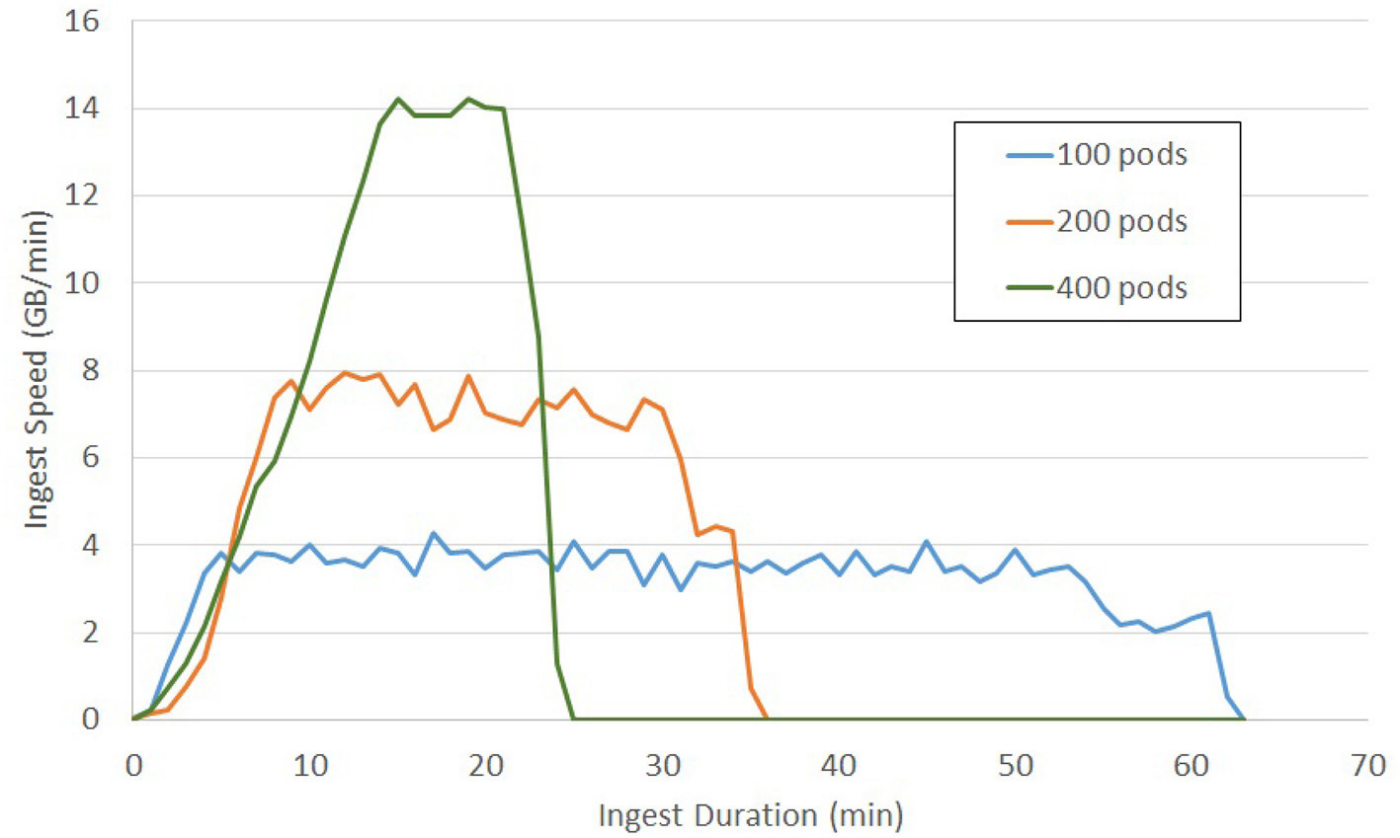
Tile Ingest throughput on demand of a 200 GB EM dataset using various scales of ingest operation.

This test shows the how BossDB will autoscale to meet demands (Figure 10). The same 3.2 million tiles from a 225-GB dataset were uploaded during each test. Each test used a different number of kubernetes pods running the ingest-client (100, 200, 400). BossDB automatically scaled endpoints, DynamoDB read and write demand to handle the throughput efficiently.

**Figure 11:**
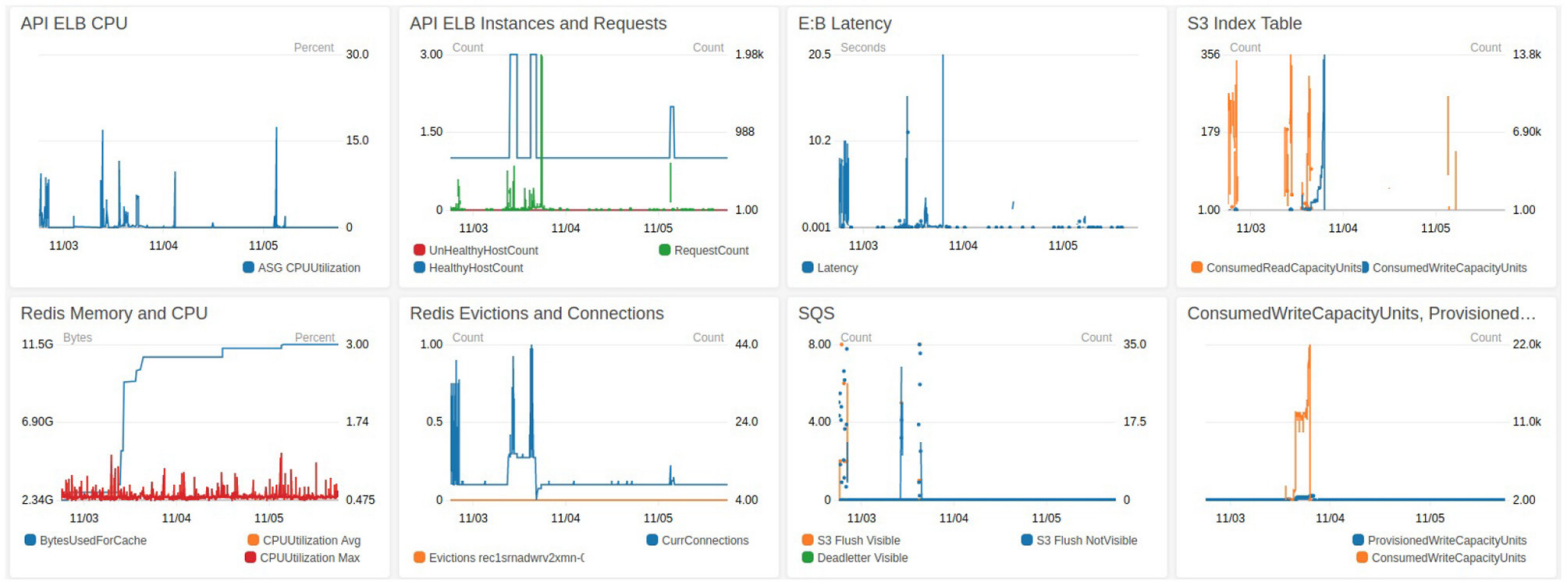
A CloudWatch dashboard monitoring during ingestion.

BossDB has monitoring capability at several levels. In Figure 11 you see a snippet of our Ingest Dashboard which allows the administrator to see how much stress any one component of the system is under. Notifications will also go out if any key components fail, and when the system hits cost milestones.

#### 3.2.4. Data Analytics

Many big data research analyses are enabled by BossDB features (e.g., standardized interfaces, arbitrary cutouts, spatial indexing), accelerating the scientific process.

One common use for BossDB is acting as a backend for local data analysis pipelines. Users download chunks of data from BossDB using intern and process it to create annotation labels using humans or machines. The resulting annotation data is uploaded via a choice of methods (python API, ingest client), below we include an example of such use case.

**Table:** 

# import intern package
from i n t e r n import a r r a y
# specify data location
COLL_NAME = 'test_collection'
EXP_NAME = 'test_experiment'
CHAN_NAME = 'test_channel'
# Use a URI to identify the data location:
chan = f''bossdb://{COLL_NAME}/{EXP_NAME}/
{CHAN_NAME}''
# Create a numpy-like pointer to the data,
# specifying the downsample-level:
d a t a s e t = a r r a y(c h a n, r e s o l u t i o n=0)
#…with access to dataset.shape,
dataset.dtype, etc.
# Download the cutout from the channel into
a 3D numpy array
d a t a = d a t a s e t[0:10, 0:512, 0:1024].

#### 3.2.5. Data Visualization and Publication

**Figure 12:**
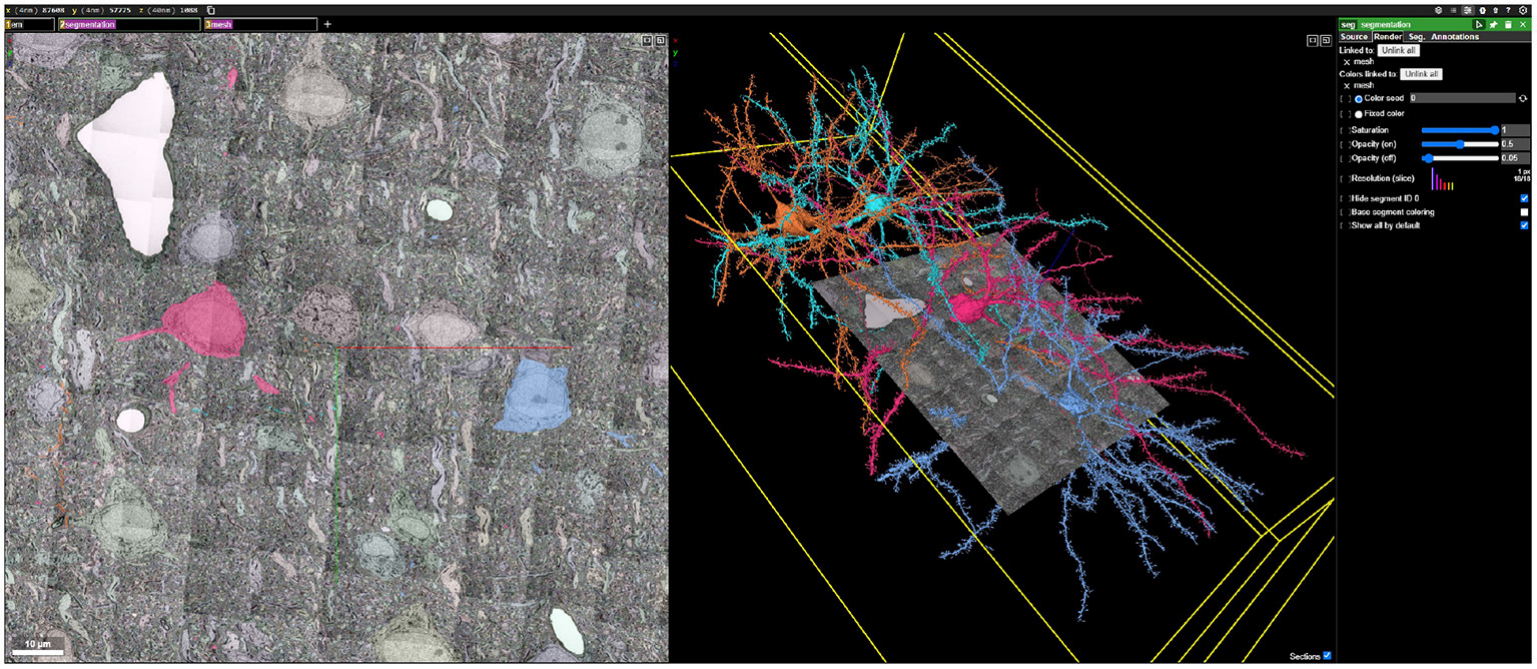
An example Neuroglancer web visualization backended by BossDB, showing public EM, segmentation, and mesh data generated during the IARPA MICrONS Program (see text footnote 4).

Data can be quickly visualized using applications such as Neuroglancer (Figure 12).

Data are published along with initial analysis, and made widely accessible through BossDB. Other research teams can then conduct additional analysis, extending and validating the existing scientific findings.

## 4. Discussion

Our data archive will enable scientists to easily access and process large datasets, and to scale up their approaches with minimal alterations and without needing large local storage. Because the results are anchored to a universally-accessible datastore, it is easier for others to inspect the results, improve upon them, and reproduce processing pipelines by leveraging common interfaces.

When considering a cloud-native approach, vendor lock-in is one potential concern – as we not only use the AWS cloud to deploy BossDB, but have integrated many of its services into the system to substantially accelerate development and performance. To minimize the development impact of expanding to an additional cloud provider or on-premise cluster, future work is needed to create a layer of abstraction between the core software and AWS services. We plan to continue to develop toward a microservices style architecture, which will decrease coupling between sub-components. This will allow BossDB to be able to independently scale sub-components and increase the ability to easily deploy, update, and manage services. We believe that storage engines will continue to specialize around datatypes (e.g., multi-dimensional image data, video data, gene sequence data) and be applicable to multiple research communities through the creation of domain-specific APIs that maintain the unique formats, organization, and needs of that community.

We intend to continue to provide BossDB as a reliable and scalable storage resource to the general microscopy and biology communities in perpetuity. We expect that as the community uses our data archive, additional tools will be developed to address new researcher needs, such as a universal, robust object-level metadata system and additional visualization engines. Several other research groups have leveraged BossDB deployments, including NeuroData (Vogelstein et al., 2018) which serves a diverse range of collaborators utilizing several imaging modalities (e.g., light microscopy, array tomography, serial multi-photon tomography) and added several new tools and capabilities to the BossDB ecosystem.

One concern about running a cloud data archive is estimating and managing cost. BossDB architecture was designed to allow dynamic scaling of resources to balance cost with performance and throughput capacity. As our software stack continues to mature, we plan to further optimize our tiered storage architecture (e.g., automatic migration data between S3 Standard, Infrequent Access, and Glacier tiers). The proposed system will provide a framework that is able to trivially scale from terabytes to petabytes while maintaining a balance between cost efficiency and performance.

As modern neuroscience datasets continue to grow in size, the community is fortunate to have several options to store and share their data. The precomputed format (Maitin-Shepard, 2021) offers a flexible, lightweight option that is readily deployable in both local and cloud settings. As mentioned above, DVID (see text footnote 1) is used to manage immutable and versioned annotations at the terascale level. We believe that our BossDB solution offers key advantages in scalability and indexing (adaptable from gigabyte to petabyte storage); authentication to manage user access workloads and costs; indexing to promote data exploration and discovery; and managed services to ensure that data is maintained and available in an efficient manner for a variety of user workflows. For a given research lab (or even within the lifecycle of a scientific question), one or more of these storage solutions may be most appropriate to enable and share results.

The standardization and scalability provided by our data archive will support a fundamental change in how researchers design and execute their experiments, and will rapidly accelerate the processing and reuse of high-quality neuroscience, most immediately for the large, petascale image, and annotation volumes produced by IARPA MICrONS. No previously existing platform met the operational and scaling requirements of the program, including managing an estimated 3–5 petabytes of image and annotation data—much larger than public neuroanatomical data archives. The BossDB software and documentation is open source and we are eager to expand the user community, supported modalities, and features. More information, examples and support are available at https://bossdb.org and https://github.com/jhuapl-boss/.

## Data Availability Statement

The original contributions presented in the study are included in the article/supplementary material, further inquiries can be directed to the corresponding author/s.

## Author Contributions

RH, DK, TG, DX, JM, DP, LR, ECJ, WG-R, and BW contributed to the BossDB system design. RH, DK, TG, DX, JM, DP, and LR contributed to software development of the BossDB system. RH, DK, DX, WG-R, JM, and BW contributed to the manuscript drafting and reviews. All authors approved the submitted version of the manuscript.

## Funding

This material is based upon work supported by the National Institutes of Health (NIH) grants R24MH114799, R24MH114785, and R01MH126684 under the NIH BRAIN Initiative Informatics Program and by the Office of the Director of National Intelligence (ODNI), and Intelligent Advanced Research Projects Activity (IARPA), via IARPA Contract No. 2017-17032700004-005 under the MICrONS program.

## Author Disclaimer

The views and conclusions contained herein are those of the authors and should not be interpreted as necessarily representing the official policies or endorsements, either expressed or implied, of the NIH, ODNI, IARPA, or the U.S. Government. The U.S. Government is authorized to reproduce and distribute reprints for Governmental purposes notwithstanding any copyright annotation therein.

## Conflict of Interest

The authors declare that the research was conducted in the absence of any commercial or financial relationships that could be construed as a potential conflict of interest.

## Publisher's Note

All claims expressed in this article are solely those of the authors and do not necessarily represent those of their affiliated organizations, or those of the publisher, the editors and the reviewers. Any product that may be evaluated in this article, or claim that may be made by its manufacturer, is not guaranteed or endorsed by the publisher.

## Footnotes

^1^Distributed, Versioned, Image-Oriented Dataservice. Available online at: https://github.com/janelia-flyem/dvid (accessed October 10, 2017).

^2^CloudVolume Is a Python Library for Reading and Writing Chunked Numpy Arrays From Neuroglancer Volumes in “precomputed” Format. Available online at: https://github.com/seung-lab/cloud-volume.

^3^Intern Software Development Kit (sdk) Tools Page on Bossdb.org. Available online at: https://bossdb.org/tools/intern (accessed December 03, 2021).

^4^MICrONS: Machine Intelligence From Cortical Networks. Available online at: http://iarpa.gov/index.php/research-programs/microns (accessed October 31, 2017).

^5^Blosc Compressor. Available online at: http://blosc.org (accessed December 03, 2021).
